# Parkinson’s disease associated with pure *ATXN10* repeat expansion

**DOI:** 10.1038/s41531-017-0029-x

**Published:** 2017-09-05

**Authors:** Birgitt Schüle, Karen N. McFarland, Kelsey Lee, Yu-Chih Tsai, Khanh-Dung Nguyen, Chao Sun, Mei Liu, Christie Byrne, Ramesh Gopi, Neng Huang, J. William Langston, Tyson Clark, Francisco Javier Jiménez Gil, Tetsudo Ashizawa

**Affiliations:** 1 Parkinson’s Institute and Clinical Center, Sunnyvale, CA 94028 USA; 2 Center for Translational Research in Neurodegenerative Disease and The McKnight Brain Institute, University of Florida, College of Medicine, Department of Neurology, Gainesville, FL 32610 USA; 3 Pacific Biosciences, Menlo Park, CA 94025 USA; 4 Biogen Idec, Cambridge, MA 02142 USA; 5 Silicon Valley Diagnostic Imaging, El Camino Hospital, Mountain View, CA 94040 USA; 6 Valley Parkinson Clinic, Los Gatos, CA 95032 USA; 7 Hospital San Javier S.A. de C.V., Guadalajara, Jalisco 44650 Mexico; 8 Houston Methodist Research Institute, Houston, TX 77030 USA

## Abstract

Large, non-coding pentanucleotide repeat expansions of ATTCT in intron 9 of the *ATXN10* gene typically cause progressive spinocerebellar ataxia with or without seizures and present neuropathologically with Purkinje cell loss resulting in symmetrical cerebellar atrophy. These *ATXN10* repeat expansions can be interrupted by sequence motifs which have been attributed to seizures and are likely to act as genetic modifiers. We identified a Mexican kindred with multiple affected family members with *ATXN10* expansions. Four affected family members showed clinical features of spinocerebellar ataxia type 10 (SCA10). However, one affected individual presented with early-onset levodopa-responsive parkinsonism, and one family member carried a large repeat *ATXN10* expansion, but was clinically unaffected. To characterize the *ATXN10* repeat, we used a novel technology of single-molecule real-time (SMRT) sequencing and CRISPR/Cas9-based capture. We sequenced the entire span of ~5.3–7.0 kb repeat expansions. The Parkinson’s patient carried an *ATXN10* expansion with no repeat interruption motifs as well as an unaffected sister. In the siblings with typical SCA10, we found a repeat pattern of ATTCC repeat motifs that have not been associated with seizures previously. Our data suggest that the absence of repeat interruptions is likely a genetic modifier for the clinical presentation of l-Dopa responsive parkinsonism, whereas repeat interruption motifs contribute clinically to epilepsy. Repeat interruptions are important genetic modifiers of the clinical phenotype in SCA10. Advanced sequencing techniques now allow to better characterize the underlying genetic architecture for determining accurate phenotype–genotype correlations.

## Medical genetics: sequence interruptions

Genetic characterization of *ATXN10* mutations reveals differences that may explain why some people develop Parkinson’s disease. The expansion of normally short sequence repeats in the *ATXN10* gene is associated with the progressive neurodegenerative disease spinocerebellar ataxia. Birgitt Schüle, at the Parkinson’s Institute and Clinical Center in Sunnyvale, California, and colleagues examined the clinical features of seven family members in a Mexican family and used the latest genetic technologies to analyze their repeat expansions in *ATXN10*. Those that experienced hallmark symptoms of spinocerebellar ataxia, such as problems with coordination and seizures, were found to carry interrupting sequences within the *ATXN10* repeat expansion. These repeat interruptions were not found in the mutated *ATXN10* sequence of one family member who developed early-onset Parkinson’s disease. Such variations in repeat expansions could determine the clinical presentation and underlying mechanism of neurodegenerative disease.

## Introduction

Spinocerebellar ataxias (SCAs) are a clinically and genetically heterogeneous group of neurodegenerative diseases with progressive cerebellar ataxia as a central pillar, but often attended by a variety of additional concomitant neurological signs and symptoms. It is now well established that 12 of these SCAs are caused by repeat expansions.^1^ The length of the repeat expansion can have an impact on age at onset, severity of symptoms, or disease progression. With more sophisticated genetic analysis tools, it is also becoming apparent that the composition of the repeat (e.g. pure expansion or an expansion that is interrupted) can modify disease phenotype.^2–4^

Spinocerebellar ataxia 10 (SCA10; OMIM#603616) is a rare autosomal-dominant progressive cerebellar syndrome. Clinically SCA10 is typically presenting first with gait ataxia followed by dysarthria, nystagmus, ocular dyskinesia. Some patients develop dementia and mood abnormalities such as depression and irritable or aggressive behavior. Focal seizures and generalized motor seizures are a common feature and usually occur several years after onset of initial symptoms. About 60% of SCA10 patients from Brazil and Mexico are reported to have seizures.^5, 6^ The average age of disease onset is 33.8 ± 9.8 years (29.5–38, *n* = 23) and it is inexorably progressive with an average disease duration of 12.9 ± 7.8 years (9.4–16.4, *n* = 23).^5^ Currently there is no available therapy except symptomatic anticonvulsive therapy for the control of seizures.

The neuropathological feature in SCA10 is Purkinje cell loss as being the primary pathological finding.^7^ The cerebellum typically demonstrates marked, symmetrical atrophy of the hemispheres, whereas the vermis is less affected. No apparent pathological changes in the cerebral cortex, hippocampus, midbrain and pons have been reported to date.^7^ Magnetic resonance imaging (MRI) scans of SCA10 patients typically show isolated cerebellar atrophy.^6, 8–13^

SCA10 cases have been primarily found in Latin American countries including Mexico,^6^ Brazil,^12^ Colombia,^14^ Argentina,^15^ Peru,^16^ Bolivia,^17^ and Venezuela,^18^ but single cases or families of SCA10 have also been reported in the USA^13^ and China.^19^ The prevalence of SCA10 among autosomal-dominant spinocerebellar syndromes has been reported highest in Mexico (13.9% reported cases)^6^ and ranges between 3 and 11.8% in Brazil^5, 12, 20^ and 4.7% in Venezuela.^18^

Expansion of an intronic ATTCT pentanucleotide repeat in the *ATXN10* gene on chromosome 22q13.31 is causative for the clinical phenotype of SCA10. Normal size of the repeat is 10–32 ATTCT, intermediate alleles (280–850 repeats) may show reduced penetrance of SCA10,^21, 22^ and full penetrance can be typically expected at 850–4500 ATTCT repeats. Repeat alleles between 33 and 279 repeats have not been reported to date. An ancestral common haplotype has been identified that is shared by all families studied to date strongly suggesting a common founder in the Amerindian population;^23^ however, there is a SCA10 family with Chinese Han ancestry recently reported with the same haplotype suggesting that the SCA10 mutation may have occurred before the divergence of Proto-Amerinds from ancestral Asians.^19^

Interestingly, several SCAs have also been associated with clinical presentation of parkinsonism. Intermediate or full disease range repeat expansions in SCA2 (*ATXN2* gene), SCA3 (*ATXN3* gene), SCA6 (*CACNA1A* gene), and SCA17 (*TBP* gene) expansions have been clinically described in patients with l-dopa responsive parkinsonism,^24^ indicating that the underlying disease process affects the dopaminergic nigrostriatal system.^25^ The majority of these SCA cases presenting with parkinsonism have an autosomal-dominant family history and the prevalence of SCA2 expansions ranges between 1 and 8% for autosomal-dominant PD.^24^ It is important to note that neuropathological case reports of patients with *ATXN2* repeat expansions have all shown alpha-synuclein positive Lewy bodies and Lewy neurites together with nigrostriatal cell loss that is highly typical of idiopathic Parkinson’s disease,^26–28^ indicating that the mutation can present in different brain regions by affecting different neuronal subtypes.

Herein, we report a rare case with an *ATXN10* repeat expansion in a patient clinically presenting with levodopa-responsive parkinsonism. Single-molecule sequencing paired with SMRT/Cas9 capture approach allowed us to characterize the genetic composition of the complete repeat expansion which revealed a novel phenotype-genotype correlation for Parkinson’s disease and *ATXN10*.

## Results

**Fig. 1:**
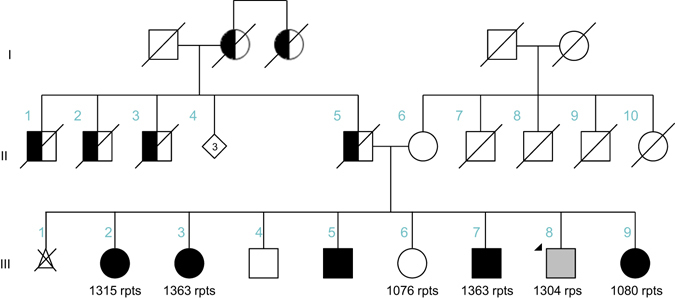
Pedigree. Four-generation pedigree of Mexican family with autosomal-dominant ataxia and Parkinson’s disease. Circles indicate female gender, squares indicate male gender, left half black symbols indicate affected individuals, but only historical information available, crossed triangle indicates miscarriage, black symbols indicate affected individuals who were also clinically evaluated by movement disorder specialist (N.H or F.J.J.G.). The gray symbol with an arrowhead indicates the proband. The SCA10 repeat size is indicated as the number of repeats (rpts)

**Fig. 2:**
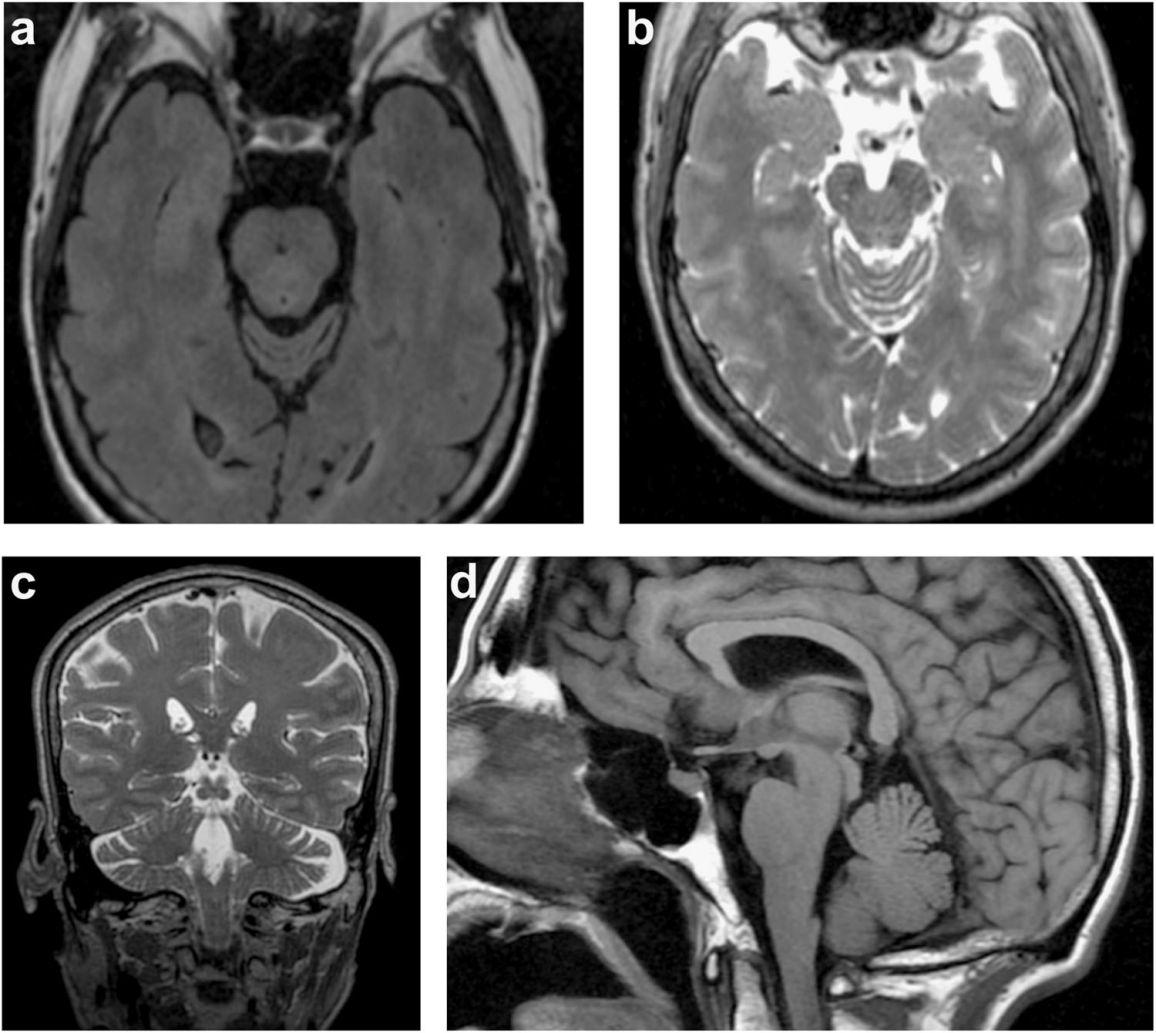
Magnet resonance imaging of the brain (Pedigree III.8). MRI was conducted as presurgical evaluation before deep brain stimulation. **a** axial FLAIR and **b** axial T2 superior vermin cistern showing mild atrophy of the folia; **c** coronal T2 illustrating expansion of extra-axial CSF containing spaces; **d** sagittal T1 shows mild superior cerebellar volume loss

We clinically and neurologically evaluated seven family members in person (by BS, NH, and FJJG). The proband, whose current age is 57 years (Pedigree III.8, Fig. 1 and Supplemental Figure), first showed clinical signs of disease at age 37, when his family noticed a “blank stare” and loss of facial expression. At 38, he developed a tremor of his chin and his left hand and generalized stiffness. At 39, his family noticed changes in his personality including increased irritability, moodiness, and generalized weakness. At age 45, he saw a neurologist who documented moderate left hand resting tremor, moderate bradykinesia, and muscle rigidity, as well as shuffling gait and balance changes. His speech was mildly soft and he exhibited moderate hypomimia. Posture was mildly stooped and his postural response on the pull test was absent. He was diagnosed as having Parkinson’s disease and responded well to dopaminergic therapy (carbidopa/levodopa and ropinirole). No cerebellar symptoms were detected. As his disease progressed, he developed motor complications with wearing off phenomenon, gait freezing, and peak-dose dyskinesia, after about 5 years of levodopa treatment. He underwent bilateral globus pallidus internus (GPi) deep brain stimulation (DBS) at age 56, approximately 17 years after onset of PD symptoms. He responded well to DBS therapy with marked relief of wearing off, gait freezing, and resolution of dyskinesia. He is currently taking levodopa (Sinemet 25/100, 2 tabs five times a day), rotigotine transdermal system (Neupro patch 6 mg/24 h), and amantadine (100 mg once a day). He is slightly bradykinetic in the morning, with hesitation in gait initiation. There is no significant wearing off on levodopa with every 3 h dosing regimen and no dyskinesia. Of note, at age 14 he had suffered a head injury, resulting in loss of consciousness for 3 h. Sense of smell (Brief smell identification test) is normal for gender and age. At age 38, he underwent surgical treatment strabismus of his right eye which was apparent since his childhood. Clinical genetic testing, which was performed (by Athena Diagnostics) because of the dominant family history, showed a repeat expansion in *ATXN10* with 1968/13 ATTCT repeats. Testing for SCA 1, 2, 3, 6, 7, 8, 14, 17 and DRPLA testing revealed normal results. An MRI scan was originally read as normal with regular appearance in volume of the brainstem including midbrain tegmentum and pons. The cerebral cortex appeared symmetric without evidence of asymmetric atrophy. There was normal T2 and susceptibility signal within the posterolateral putamen without evidence of abnormal iron accumulation. There were no structural signs of atypical parkinsonian syndrome noted. After the *ATXN10* expansion mutation was identified, the scan was re-evaluated, and it appeared that there were some mild expansion of the folia and some prominence of the extra-axial cerebrospinal fluid containing spaces of the posterior fossa which suggested mild cerebellar atrophy, but could also be age-related (Fig. 2).

Symptom onset in the proband’s father (Pedigree II.5) was at age 35 with progressive ataxia. He developed seizures at age 66, but had no reported mental deterioration. He died at age 67 from a myocardial infarction. No clinical records of neurological findings or biospecimens are available for analysis or interpretation. In the mother (Pedigree II.6) no signs or symptoms of parkinsonism or ataxia were noted at age 90 on clinical and neurological evaluation by F.J.J.G. The oldest sister 1 (Pedigree III.2) reported an age at onset at age 35 years with typical progressive SCA. Focal motor seizures with impaired awareness started at age 60 years. The second oldest sister 2 (Pedigree III.3) had an age at onset at age 48 years also with typical progressive SCA. At age 57, she also developed focal motor seizures with impaired awareness. Sister 3 (Pedigree III.6) showed clinically no parkinsonian symptoms of resting or action tremor, rigidity, or bradykinesia at 61 years of age. No gait abnormalities were observed with normal stride and tandem gait. No abnormalities in heel-shin or finger chase test were noted on clinical neurological evaluation by F.J.J.G. The proband’s affected brother (Pedigree III.7) developed symptoms at age 49 years with typical progressive SCA and started experiencing focal seizures at age 53 years. The youngest sister 4 (Pedigree III.9) first developed symptoms at age 37 with typical progressive spinocerebellar ataxia. She did not experience seizures at time of evaluation.

A summary of key clinical features is listed in Table 1 for all individuals with detected *ATXN10* repeat expansions. Additional detailed clinical information for the four siblings with SCA10 spinocerebellar ataxia is described in the supplemental material.

**Table 1:** Clinical features

	III.2	III.3	III.6	III.7	III.8 (Proband)	III.9
Lab ID	PI-2568	PI-2569	PI-2570	PI-1202	PI-1124	PI-2571
Gender	F	F	F	M	M	F
*ATXN10* repeat (SMRT/Cas9 capture)*	1315	1363	1076	1363	1304	1080
Age at onset (years)	35	48	unaffected	48	38	37
Onset of seizures (years)	60	57	–	53	–	–
Current age (years)	71	68	61	65	57	53
Duration of disease (years)	36	20	–	17	19	16
Seizures	+	+	–	+	–	–
Gait ataxia	+	+	–	+	–	+
Intention tremor	+	+	–	–	+	–
Dysarthria	+	+	–	+	–	+
Dysmetria	+	+	–	+	–	+
Dysdiadochokinesia	+	+	–	–	+	+
Nystagmus	+	+	–	+	–	+
Ocular dyskinesia	+	+	–	+	–	–
Hypotonia	–	–	–	–	–	–
Hyperreflexia	–	–	–	+	–	–
Babinski’s sign	–	–	–	–	–	–
Leg spasticity	–	–	–	–	–	–
Aggressiveness	+	+	–	–	–	+
Depression	+	+	–	+	+	+
MoCA	9/30	2/30	29/30	U	U	25/30
SARA	23.5/40	24.5/40	0/40	U	6/40	16/40
B-SIT (% tile)	NP	NP	27%	U	29%	20%
B-SIT (category)	NP	NP	Normal	U	Normal	Normal

*B-SIT* Brief Smell Identification Test, *F* female, *M* male, *MoCA* Montreal cognitive assessment, *NP* not possible: patient was not able to perform test, *SARA* Scale for Assessment and Rating of Ataxia, *U* unknown, *+* symptom present, *−* symptom not present, * the shortest circular consensus (ccs) read

**Fig. 3:**
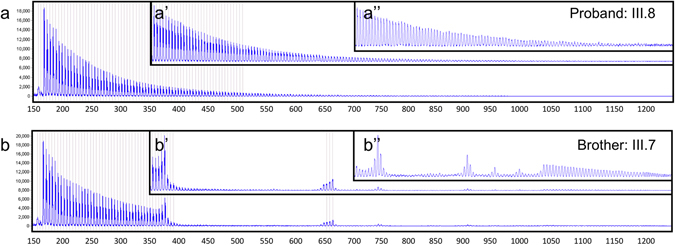
XL-repeat-primed PCR. Repeat-primed PCR (RP-PCR) products separated on a 50-cm capillary. PCR products begin around 160 bp and occur at 5 bp intervals except at the location of repeat interruption (*y*-axis: signal strength; *x*-axis product size in bp). **a** RP-PCR products from the proband (III.8) indicate a regular 5 bp ladder through the entire length of resolution (1250 bp). **b** RP-PCR products from his affected brother (III.7) display multiple interruptions in ATTCT peaks between ~350 and 650 bp, ~675 and 725 bp, ~~750 and 900 and smaller areas between 900 and 1050 bp. The regular ATTCT ladder continues from 1050 bp through the length of resolution (1250 bp). Insets are shown at a higher resolution between 350–1250 (a′, b′) and 700–1250 bp (a″, b″)

To further genetically characterize the *ATXN10* repeat expansion and to better understand the phenotypic differences of progressive cerebellar ataxia with seizures and parkinsonism, we employed several advanced and novel molecular genetic techniques to dissect the genetic structure of the repeat expansion in this family. First, repeat-primed PCR (RP-PCR) was performed which indicated the presence of an expanded allele in both the proband and his affected brother. RP-PCR relies on the annealing of a repeat-primer to the expansion allele which produces a regular 5-bp “ladder” of PCR products separated on capillary electrophoresis (Fig. 3).^29^ This technique not only detects the presence of an expansion allele by the extension of the ladder beyond the range of normal alleles, but is also useful for detecting the presence of interruption motifs within the first ~1250 bp of the 5′ end of the expansion. The RP-PCR pattern in the proband (Fig. 3, III.8) shows the regularly spaced, 5 bp intervals. However, the RP-PCR pattern in his affected brother (Fig. 3, III.7), who presented with ataxia and epilepsy, displayed multiple gaps in the ladder of peaks indicating the presence of multiple, complex interruption motifs. This interruption motif of the 5′ region of the expansion detected by repeat-primed PCR has been observed in other SCA10 families (K. McFarland, personal communication).

**Fig. 4:**
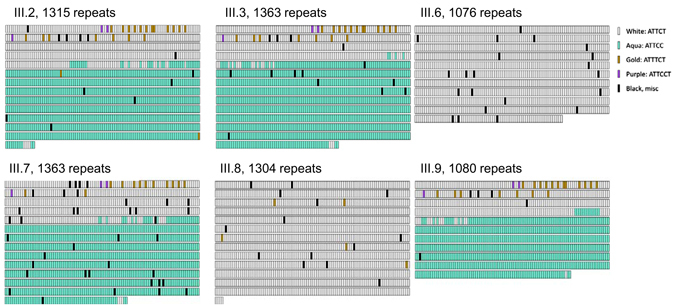
ATXN10 repeat expansion sequence schematics based on PacBio sequencing. Repeat expansions are represented in the 5′ (upper left) to 3′ (lower right) direction. The schematics and expansion size given are based on the shortest most error-free read length for each individual and family members in the pedigree as follows: III.2: 1315 repeats, III.3: 1363 repeats, III.6: 1076 repeats, III.7: 1363 repeats, III.8: 1304 repeats and III.9: 1080 repeats. Each rectangle represents a sequence motif as follows: white, ATTCT; aqua, ATTCC; gold, ATTTCT; purple, ATTCCT. Black rectangles denote miscellaneous motifs that may represent errors in sequence reads

Next, we applied a novel SMRT/Cas9 capture approach paired with third-generation sequencing that allowed us to sequence up to 1400 repeat motifs or 7 kb of sequence for six family members as one continuous fragment without prior amplification of the genomic DNA (Fig. 4). Four sisters and two brothers of this family gave consent for genetic analysis. We detected a ~1400 repeat pattern that is composed of approximately 2.4 kb/480 repeats with the typical ATTCT pentanucleotide repeat motif followed by approximately 4.6 kb/920 repeats with an ATTCC pentanucleotide repeat motif in all individuals with clinical presentation of cerebellar ataxia and seizures (Fig. 4). Interestingly, the “common” ATTCT pentanucleotide repeat motif only made up about 40% of the total repeat expansion, whereas the “repeat interruption” motif ATTCC contributed to approximately 60% of the size of the expansion.

On the contrary, in our patient with early-onset levodopa-responsive parkinsonism we identified a slightly smaller *ATXN10* repeat expansion of 1304 ‘common’ ATTCT repeats without the pentanucleotide interruptions ATTCC (Fig. 4, III.8). In addition, one sister who also carried the repeat expansion of ~1076 pure ‘common’ ATTCT repeats does not show any clinical symptoms of parkinsonism or ataxia at age 61 (Fig. 4, III.6).

To summarize the group characteristics and clinical features, the average age at onset in this large Mexican family with phenotypes of autosomal-dominant progressive cerebellar ataxia and one case of clinically diagnosed Parkinson’s disease was 40.5 years (range 35–49 years, SD 6.28 years) and the disease duration was 21.6 years (range 16–38 years, SD 8.20). Affected siblings (III. 2, 3, 7, 9) with cerebellar ataxia exhibited scanning speech, oculomotor ataxia with hypermetric saccades, moderate to severe cognitive decline, depression and anxiety as well as aggressive behavior (Table 1). The disease in this family typically started with lower limb ataxia and gait disturbances followed by upper limb ataxia. Three of the five affected family members also suffer from epilepsy occurring 4–25 years after the onset of ataxia and presenting as focal motor seizures with impaired awareness (III.2, 3, 7) that can generalize to an epileptic crisis (Table 1).

In contrast, the proband (Pedigree III.8) was clinically diagnosed with Parkinson’s disease starting at age 37. On the neurological examination, no clear signs of cerebellar ataxia were noted and the re-evaluation of his MRI (RG) showed very mild cerebellar atrophy indicating that the neuropathological process might affect both nigrostriatal system and cerebellum (Fig. 2), but neuroradiologically this could also be part of a normal aging process. The patient developed typical levodopa-induced long-term side effects of dyskinesias and underwent bilateral GPi DBS with significant improvement of the dyskinesias. Interestingly, the smell identification test was within normal limits, which is also reported for other genetic forms of Parkinson’s disease, e.g. PARK2.^30, 31^

In addition, we also identified one of the siblings (Pedigree III.6) at age 61 who did not show any clinical signs of ataxia or parkinsonism, or other neurological features, who proved to have a very similar repeat expansion without repeat interruptions as the proband with Parkinson’s disease. The repeat expansion presented in this unaffected sibling consists of 1076 repeats. On examination, she had a normal sense of smell; however, imaging studies (e.g. DATscan) were not possible due to the remote location this family lives. She has passed the mean age at onset in this family by more than 20 years. Her case suggests that this type of ‘pure’ repeat expansion can also show reduced or delayed penetrance. Other shorter *ATXN10* expansions have been reported with reduced penetrance up to 850 pentanucleotide repeats.^21, 22^ One SCA10 case (subject A^32^) has been described with an age at onset of ataxia and non-levodopa-responsive parkinsonian features at 83 and 1400 ATXN10 repeat expansions;^13, 32^ therefore, we cannot exclude that this sibling might be presymptomatic and might develop symptoms of SCA or parkinsonian at a later age.

The most important aspect of this study is that the molecular genetic characterization of the repeat motif composition at the 5′ end of the expansion by RP-PCR (and of the entire expansion allele via SMRT/Cas9 capture third-generation sequencing) revealed differences in the SCA10 expansion between siblings. The proband who presented with clinically diagnosed Parkinson’s disease had a highly ‘pure’ ATTCT expansion allele with few (if any) interruption motifs present. This expansion is the ‘purest’ of *SCA10* repeat expansions to be examined and sequenced to date.

Furthermore, the proband’s affected brother and three sisters with ataxia, epilepsy, and cognitive impairment had a stable highly interrupted SCA10 expansion, consisting of 40% ‘common’ ATTCT repeats in the 5′ end and 60% ATTCC repeats in the 3′ end. Thus, the ATTCC repeats were either lost in the proband and unaffected sibling or gained in affected siblings with SCA10 during the transmission of the allele from their affected father. It is also unclear whether this loss or gain of the large ATTCC repeat occurred in the father’s germline or in the offspring’s soma, and whether or not it occurred through step-wise loss or gain of ATTCC repeat units or an insertion/deletion/conversion of a long ATTCC repeat tract. The father died at age 67 and had cerebellar ataxia. He only developed seizures a year before his death at age 66. Since the phenotype in the father is similar to the affected siblings with SCA10, we propose that the father also carried the complex repeat interruption.

## Discussion

We describe a rare case with clinically diagnosed Parkinson’s disease and an *ATXN10* repeat expansion. Genetic characterization of the repeat expansion revealed that the sibling with Parkinson’s disease had a different *ATXN10* repeat pattern, which did not include repeat interruptions compared to his siblings with typical SCA10. We propose that the absence of repeat interruptions play a role in the underlying disease process acting as a genetic modifier and leading to the clinical presentation of l-Dopa-responsive parkinsonism. These findings expand the allelic spectrum of mutations in the *ATXN10* gene and emphasize the role of genetic modifiers, in this case the composition of the *ATXN10* repeat expansion.

Repeat interruptions of the expanded *ATXN1*0 allele have been attributed to seizures. Generalized motor seizures and/or complex partial seizures have been reported in families from Mexico (60%, 24 of 60) and Brazil (3.75%, 3 of 80). It has been calculated that the occurrence of ATCCT interruptions within the *ATXN10* ATTCT expansion leads to a 6.3-fold increased risk of developing epilepsy in patients with cerebellar ataxia.^3^ The repeat interruption in our family is different and comprises an ATTCC interruption. This ATTCC interruption has not been previously associated with seizures in other families. A similar ATTCC interruption has been described (subject B^32^), but without occurrence of seizures.^32^ In our family, three of the four genetically characterized SCA10 patients also presented with seizures carried the ATTCC interruption motif (Fig. 4, III.2, 3, 7). Thus, the ATTCC interruption is likely a novel genetic modifier for the clinical occurrence of seizures in SCA10.

It will be interesting to understand how different *ATXN10* repeat expansions with and without repeat interruptions can lead to different clinical phenotypes resulting from differential neuronal vulnerability of dopamine neurons in the substantia nigra and the GABAergic Purkinje cells as suggested by the finding in this kindred. Both neuronal subtypes have large dendritic arborization with thousands of synapses that have a high energy demand which could lead to a preferential vulnerability. Future studies are needed to understand how different compositions of *ATXN10* repeat interruptions produce selective neuronal neurodegeneration. Human pluripotent stem cell-derived neuronal cultures from these patients and families might allow us to further investigate these critical questions and to develop targeted therapeutic approaches.^33^

There are several key findings highlighting the importance for complete sequencing of repeat expansions to unravel genetic modifiers of clinical phenotypes. First, we expand the phenotypic spectrum of the *ATXN10* repeat expansion and report a patient with clinically defined Parkinson’s disease. Second, the *ATXN10* expansion in this case with Parkinson’s disease has virtually no repeat interruptions and presents the ‘purest’ repeat reported to date. Third, this same pure repeat expansion was also found in an unaffected sibling, supporting this also as a reduced penetrance allele. Fourth, the *ATXN10* repeat expansion in four siblings with cerebellar ataxia, seizures, and progressive dementia had an unusual repeat interruption of ATTCC, encompassing about 60% of the overall repeat expansion, which has not been reported to be associated with seizures.

In summary, we conclude that the underlying genetic architecture of *ATXN10* repeat expansions is critical for presentation of clinical phenotypes and presumably also the underlying pathology.

## Methods

Blood samples or saliva were collected after obtaining informed consent from each participant approved by the Institutional Review Board at El Camino Hospital, Mountain View, CA (Protocol ECH-99-22). Methods were performed in accordance with relevant regulations and guidelines. Genomic DNA was isolated from peripheral blood leukocytes.^34^ DNA from saliva was isolated using Oragene Discover (OGR-500, DNA Genotek) collection kits in combination with PrepIT L2P (DNA Genotek) extraction.

To exclude causative mutations or risk alleles in other genes related to Parkinson’s disease, amyotrophic lateral sclerosis, and Alzheimer disease, we sequenced all exons, promoters, and exon–intron boundaries for 188 genes (Supplemental Table). We used Agilent SureSelect for target enrichment for the genes on a next-generation sequencing platform. The raw data were aligned using GATK workflow for pre-processing, variant discovery, and call-set refinement.^35^ After variant processing, sequence variants were annotated using Variant Effect Predictor toolkit.^36^ We did not detect any sequence variants in coding or known regulatory regions (promoter or splice-site junctions) that we predicted to be causative and contributing to the clinical phenotype.

To determine the composition of the repeat expansion, we performed RP-PCR as described^29^ with modifications as follows. One hundred nanograms of genomic DNA was used as a template in a 25 μl PCR reaction containing primers P1 (5′-GAAGACAAATAGAAAACAGATGGCAGA-3′), P2B (5′-TACGCATCCCAGTTTGAGACGG(AATAG)_8_-3′), and tail (5′-TACGCATCCCAGTTTGAGACGG-3′) using AmpliTaq Gold 360 polymerase master mix (Applied Biosciences). Primer P1 was FAM-labeled and primers were added to a final concentration of 0.4 nM (P1 & tail) and 0.04 nM (P2). Products were amplified under cycling conditions: 95^o^C, 10 min, initial denaturing; followed by 35 cycles of: 95^o^C, 15 s; 61^o^C, 30 s; 72^o^C, 5 min with a final extension time of 72^o^C for 10 min. Samples were submitted for fragment analysis on the ABI PRISM 3730 XL at the genotyping core at the University of Florida’s Interdisciplinary Center for Biotechnology Research (ICBR). For fragment analysis, samples were run (without dilution) with a 50 cm capillary to allow for product size differentiation of up to 1200 bp.

To assess the full length of the repeat we employed a novel SMRT/Cas9 capture method combined with third-generation sequencing. Blood or saliva genomic DNA was digested with *Eco*RI–*Bam*HI to attach SMRTBell.^37^ The crRNA for Cas9 digestion has a 20nt sequence complementary to DNA target (ATACAAAGGATCAGAAT_CCC) and the cut occurs three bases upstream from the 3′ end of this 20 base sequence (between T and C) just upstream of the repeat region. A capture adapter was ligated to the Cas9 double-strand cleavage site and pulled down using MagBeads that recognize the capture adapter. The MagBead-enriched DNA fragments that contain SCA10 repeats are subjected to SMRT sequencing.

### Data availability statement

Fasta files for ATXN10 repeat expansions are available upon request from corresponding author.

The accession number for the ATXN10 gene reported in this paper is NM_013236.3 and gene locus MIM number is 611150.

## Electronic supplementary material

Supplemental Material

## References

[1] Sun YM, Lu C, Wu ZY (2016). Spinocerebellar ataxia: relationship between phenotype and genotype—a review. Clin. Genet.,.

[2] Menon RP (2013). The role of interruptions in polyQ in the pathology of SCA1. PLoS Genet..

[3] McFarland KN (2014). Repeat interruptions in spinocerebellar ataxia type 10 expansions are strongly associated with epileptic seizures. Neurogenetics.

[4] Wang C (2015). Linkage analysis and whole-exome sequencing exclude extra mutations responsible for the parkinsonian phenotype of spinocerebellar ataxia-2. Neurobiol. Aging.

[5] de Castilhos RM (2014). Spinocerebellar ataxias in Brazil--frequencies and modulating effects of related genes. Cerebellum.

[6] Rasmussen A (2001). Clinical and genetic analysis of four Mexican families with spinocerebellar ataxia type 10. Ann. Neurol..

[7] Xia G (2013). Purkinje cell loss is the major brain pathology of spinocerebellar ataxia type 10. J. Neurol. Neurosurg. Psychiatry.

[8] Grewal RP (1998). Clinical and genetic analysis of a distinct autosomal dominant spinocerebellar ataxia. Neurology.

[9] Matsuura T (1999). Mapping of the gene for a novel spinocerebellar ataxia with pure cerebellar signs and epilepsy. Ann. Neurol..

[10] Zu L, Figueroa KP, Grewal R, Pulst SM (1999). Mapping of a new autosomal dominant spinocerebellar ataxia to chromosome 22. Am. J. Hum. Genet..

[11] Matsuura T (2000). Large expansion of the ATTCT pentanucleotide repeat in spinocerebellar ataxia type 10. Nat. Genet..

[12] Teive HA (2004). Clinical phenotype of Brazilian families with spinocerebellar ataxia 10. Neurology.

[13] Bushara K (2013). Expansion of the Spinocerebellar ataxia type 10 (SCA10) repeat in a patient with Sioux Native American ancestry. PLoS ONE.

[14] Roxburgh RH (2013). The unique co-occurrence of spinocerebellar ataxia type 10 (SCA10) and Huntington disease. J. Neurol. Sci..

[15] Gatto EM (2007). Ethnic origin and extrapyramidal signs in an Argentinean spinocerebellar ataxia type 10 family. Neurology.

[16] Leonardi L (2014). Spinocerebellar ataxia type 10 in Peru: the missing link in the Amerindian origin of the disease. J. Neurol..

[17] Baizabal-Carvallo JF (2015). Bolivian kindred with combined spinocerebellar ataxia types 2 and 10. Acta Neurol. Scand..

[18] Paradisi I, Ikonomu V, Arias S (2016). Spinocerebellar ataxias in Venezuela: genetic epidemiology and their most likely ethnic descent. J. Hum. Genet..

[19] Wang K (2015). Spinocerebellar ataxia type 10 in Chinese Han. Neurol. Genet.

[20] Teive HA (2012). Spinocerebellar ataxias: genotype-phenotype correlations in 104 Brazilian families. Clinics (Sao Paulo).

[21] Alonso I (2006). Reduced penetrance of intermediate size alleles in spinocerebellar ataxia type 10. Neurology.

[22] Raskin S (2007). Reduced penetrance in a Brazilian family with spinocerebellar ataxia type 10. Arch. Neurol..

[23] Almeida T (2009). Ancestral origin of the ATTCT repeat expansion in spinocerebellar ataxia type 10 (SCA10). PLoS ONE.

[24] Park H, Kim HJ, Jeon BS (2015). Parkinsonism in spinocerebellar ataxia. BioMed. Res. Int..

[25] Braak H, Del Tredici K (2009). Neuroanatomy and pathology of sporadic Parkinson’s disease. Adv. Anat. Embryol. Cell. Biol..

[26] Takao, M. et al. Spinocerebellar ataxia type 2 is associated with Parkinsonism and Lewy body pathology. *BMJ Case Rep.* **2011**, 10.1136/bcr.01.2011.3685 (2011). 10.1136/bcr.01.2011.3685 PMC3079476 22700602

[27] Rub U (2013). Clinical features, neurogenetics and neuropathology of the polyglutamine spinocerebellar ataxias type 1, 2, 3, 6 and 7. Prog. Neurobiol..

[28] Langston, J. W., Schüle, B., Rees, L., Nichols, R. J. & Barlow, C. Multisystem Lewy body disease and the other parkinsonian disorders. *Nat. Genet.* **47**, 1378–1384 (2015). 10.1038/ng.3454 26620112

[29] McFarland KN (2013). Paradoxical effects of repeat interruptions on spinocerebellar ataxia type 10 expansions and repeat instability. Eur. J. Hum. Genet..

[30] Malek N (2016). Olfaction in Parkin single and compound heterozygotes in a cohort of young onset Parkinson’s disease patients. Acta Neurol. Scand..

[31] Alcalay RN (2011). Olfaction in Parkin heterozygotes and compound heterozygotes: the CORE-PD study. Neurology.

[32] McFarland KN (2015). SMRT sequencing of long tandem nucleotide repeats in SCA10 reveals unique insight of repeat expansion structure. PLoS ONE.

[33] Yang WY, Gao R, Southern M, Sarkar PS, Disney MD (2016). Design of a bioactive small molecule that targets r(AUUCU) repeats in spinocerebellar ataxia 10. Nat. Commun..

[34] Miller SA, Dykes DD, Polesky HF (1988). A simple salting out procedure for extracting DNA from human nucleated cells. Nucleic Acids Res..

[35] McKenna A (2010). The genome analysis toolkit: a MapReduce framework for analyzing next-generation DNA sequencing data. Genome Res..

[36] McLaren W (2010). Deriving the consequences of genomic variants with the Ensembl API and SNP Effect Predictor. Bioinformatics.

[37] Travers KJ, Chin CS, Rank DR, Eid JS, Turner SW (2010). A flexible and efficient template format for circular consensus sequencing and SNP detection. Nucleic Acids Res..

