# Supplementary material for: Parkinson’s disease associated with pure ATXN10 repeat expansion
Source: NPJ Parkinsons Dis. 2017 Sep 5;3:27. doi: 10.1038/s41531-017-0029-x (PMC5585403; doi:10.1038/s41531-017-0029-x)

**Supplemental Data.**

**Supplemental data include detailed description of clinical case histories, a table, and a figure of the extended pedigree.**

**Clinical case histories:**

**Sister 1 (Pedigree III.2):** At age 35 years this patient first noticed a change in her walking which was described as “marching like a soldier”. Five years later, the she began experiencing uncoordinated movements and her speech became increasingly garbled and explosive. The patient has used a walker since age 53 and can walk distances of 5-9 meters with the walker but needs support from her family to walk longer distances or leave her home. At age 60, she started experiencing complex partial seizures and also started experiencing diplopia in all directions of gaze. Her speech has become intelligible. She denies numbness or tingling of her hands or feet. She has also experienced mild memory loss for seven years. She smoked briefly in her 20s, and had no history of drinking alcohol or using recreational drugs. Recently she experienced a generalized tonic-clonic seizure. She has not taken any medications, although she had previously used captopril and valproate. Currently, the patient has severe cognitive changes (MOCA 9/30), mild depression, anxiety, and apathy. At neurologic examination, the patient showed a conjugate gaze palsy with endotropia on the right side and tracking ocular ataxia with hypometric saccades of the right and hypermetric saccades of the left eye. The patient showed hypermetria on both sides during a finger-nose test and very severe dysmetria during heel-shin testing bilaterally. There was no postural, rest, or simple kinetic tremor although there was intention tremor). Her gait was markedly ataxic, and speech was explosive and dysarthric. Her radial, bicipital and radial reflexes were 2+, patellar reflex was 3+ and Achilles reflex was 1+, Babinski sign was absent. The total SARA rating score was 23.5/40 (Gait - 7; Stance - 5; Sitting - 1; Speech disturbance - 3; Finger Chase R - 1; Finger Chase L - 1; Nose-finger test R - 2; Nose-finger test L - 2; Fast alternating hand movements R - 2; Fast alternating hand movements L - 2; Heel-shin slide R - 4; Heel-shin side L - 4).

**Sister 2 (Pedigree III.3):** The patient's first symptoms began at age 48 when she noticed problems with walking and occasional falls. Over the next 9 years she developed incoordination of her hands and legs and marked speech difficulties. At age 57, she developed complex partial seizures with secondary generalization. She currently has seizures daily, usually early in the morning, but these are mild in intensity and short in duration. She reports having cold feet, but denies numbness or tingling of her hands or feet. She has mild depression and anxiety. She also experienced progressive memory loss since her late 50’s and does not recognize the majority of her family members. She is physically aggressive and hits family members. She is completely dependent on her family.

Her current examination reveals a severe cognitive impairment (MOCA 2/30). She has bilateral hypermetric saccades and hypermetria in both hands. Total SARA rating score was 24.5/40 (Gait - 8; Stance - 5; Sitting - 1; Speech disturbance - 1; Finger Chase R - 2; Finger Chase L - 2; Nose-finger test R - 3; Nose-finger test L - 3; Fast alternating hand movements R - 1; Fast alternating hand movements L - 2; Heel-shin slide R - 4; Heel-shin side L - 4). The patient does not have rigidity or postural, rest, or kinetic tremor. She currently takes carbamazepine and levetiracetam for her seizures.

**Brother (Pedigree III.8)**: The brother of the proband began noting problems with coordination at 48 years of age, manifested as an unsteady gait, and a gradual worsening sense of imbalance. This was followed by generalized weakness, fatigue, speech difficulties, depression, and cognitive disturbance. His everyday living became slower and more difficult and he developed trouble with both his short and long term memory. He developed seizures at 53 years old. He was seen by NH at age 53, who diagnosed him with cerebellar ataxia based upon the presence of nystagmus, dysarthria, limb ataxia, and gait ataxia, in the absence of parkinsonism. Genetic testing (Athena Diagnostics) showed a repeat expansion in *ATXN10* with 2223/13 ATTCT repeats. Testing for SCA 1, 2, 3, 6, 7, 8, 14, 17, and DRPLA testing revealed normal results.

**Sister 4 (Pedigree III.10):** This 53-year-old female first developed problems at age 37, immediately after giving birth to her last child. Her feet became "clumsy," she could no longer walk quickly or run, had difficulty dancing. Over the next 16 years she experienced occasional diplopia and began using a walker even for short distances. She had progressive memory loss during this period of time. Over the last three years she had become more physically aggressive, but never experience hallucinations. When examined by FJJG, she was disoriented in time, but knew where she was. She was friendly and cooperative during examination. No seizures reported. MOCA score was 24/30 (received extra point for education less than 12 yrs). She had explosive dysarthria (2/4) and strabismus, hypermetric dysmetric movements (2/4) in the arms. Her total SARA rating score was 18/40 points (Gait - 6; Stance - 2; Sitting - 0; Speech disturbance - 4; Finger Chase R - 1; Finger Chase L - 1; Nose-finger test R - 1; Nose-finger test L - 1; Fast alternating hand movements R - 1; Fast alternating hand movements L - 2; Heel-shin slide R - 2; Heel-shin side L - 3). She is not taking medication except for dietary supplements.

**Supplemental table.**

**List of SCA repeat expansions and 188 genes sequenced to exclude other causative mutations in genes related to Parkinson’s disease or neurodegeneration**

| **SCA repeat expansion and mutation analysis** | | | | | | |
| --- | --- | --- | --- | --- | --- | --- |
| **Gene name** | **Chrom.** |  |  | **Gene name** | **Chrom.** |  |
| ATXN1 | 6p22.3 |  |  | ATXN8 | 13q21 |  |
| ATXN2 | 12q24.12 |  |  | ATXN17 | 6q27 |  |
| AXTN3 | 14q32.12 |  |  | PRKCG | 19q13.42 |  |
| ATXN6 | 19p13.13 |  |  | DRPLA | 12p13.31 |  |
| ATXN7 | 3p14.1 |  |  |  |  |  |
| **188-gene panel for neurodegenerative diseases** | | | | | | |
| **Gene name** | **Chrom.** | **Gene Range** |  | **Gene name** | **Chrom.** | **Gene Range** |
| APOE | 19 | 45409039..45412650 |  | TMEM43 | 3 | 14124940..14143680 |
| APP | 21 | 27252861..27543446 |  | TTYH3 | 7 | 2631969..2664802 |
| PSEN2 | 1 | 227058273..227083804 |  | FAM73B | 9 | 129036620..129072082 |
| TREM2 | 6 | 41126246..41130922 |  | NAV2 | 11 | 19350724..20121601 |
| ANG | 14q11.2 | 21152336..21162345 |  | TMEM67 | 8 | 93754844..93819234 |
| C21orf2 | 21 | 44328944..44339402 |  | ACBD5 | 10 | 27195214..27242275 |
| CAMK2A | 5 | 150219491..150289840 |  | VPS26B | 11 | 134224667..134247792 |
| DAO | 12 | 108880030..108900934 |  | CYGB | 17 | 76527348..76537905 |
| DCTN1 | 2 | 74588281..74619214 |  | OSBPL1A | 18 | 24162045..24397882 |
| DDX6 | 11 | 118747763..118791263 |  | OSBPL6 | 2 | 178194481..178399433 |
| DNMT3A | 2 | 25228552..25342590 |  | OSBPL9 | 1 | 51616874..51789219 |
| FUS | 16p11.2 | 31191431..31206192 |  | OSBPL10 | 3 | 31660825..31981850 |
| HFE | 6 | 26087509..26095469 |  | OSBPL11 | 3 | 125528858..125595537 |
| hnRNPA1 | 12 | 54280696..54287087 |  | ZNF837 | 19 | 58367623..58381022 |
| hnRNPA2B1 | 7 | 26189927..26200793 |  | FRA10AC1 | 10 | 93667883..93702572 |
| LCN 2 | 9 | 128149453..128153455 |  | FAM82A1 | 2 | 37925319..38067142 |
| NEFH | 22 | 29876181..29887279 |  | MBOAT1 | 6 | 20099684..20212464 |
| NEK1 | 4 | 169393270..169612629 |  | VPS13B | 8 | 99011179..99878219 |
| NSF | 17 | 46590669..46757464 |  | TTC39B | 9 | 15170844..15307360 |
| OSBP | 11 | 59574398..59616144 |  | MOSPD2 | X | 14873405..14922166 |
| PRODH | 22 | 18912774..18936553 |  | ZNF296 | 19 | 45071500..45076430 |
| PRPH | 12 | 49688909..49692481 |  | C9ORF72 | 9p21.2 | 27546544..27573842 |
| PYGM | 11 | 64746389..64760715 |  | GRID2IP | 7 | 6496778..6551436 |
| RAB2A | 8 | 60516910..60623644 |  | MAPT | 17q21.31 | 43971748..44105700 ?? |
| ATXN1 | 6 | 16299112..16761490 |  | PSEN1 | 14q24.2 | 73603143..73690399 |
| ATXN2 | 12q24.12 | 111890018..112037480 |  | TRPM7 | 15q21.2 | 50849351..50979012 |
| SDF2 | 17 | 28648356..28662189 |  | AFF2 | X | 148500619..149000663 |
| SLC1A5 | 19 | 46760328..46789019 |  | EPHA4 | 2 | 221418027..221572290 |
| SOD1 | 21 | 33031935..33041244 |  | USP14 | 18 | 158483..213739 |
| SPAST | 2 | 32063592..32157637 |  | HDAC6 | X | 48660487..48683380 |
| TFRC | 3 | 196049284..196082161 |  | PFN1 | 17 | 4848945..4852381 |
| TIAL1 | 10 | 119573465..119597029 |  | APC | 5 | 112707505..112846239 |
| TTC1 | 5 | 160009100..160065545 |  | BST1 | 4 | 15702950..15739498 |
| UBE2D2 | 5 | 139561166..139628434 |  | CALM1 | 14 | 90396983..90408275 |
| VCP | 9 | 35056065..35072739 |  | CALM3 | 19 | 46601255..46610782 |
| TAF15 | 17 | 35809455..35847242 |  | SCARB2 | 4 | 76158737..76213899 |
| PABPC4 | 1 | 39560813..39576849 |  | CDK3 | 17 | 76000906..76005999 |
| VAPB | 20q13.32 | 56964175..57026157 |  | CHRNA3 | 15 | 78593052..78621295 |
| FADS2 | 11 | 61816203..61867354 |  | CSNK1E | 22 | 38290691..38318084 |
| EIF2AK3 | 2 | 88556741..88627576 |  | CSNK1G3 | 5 | 123512099..123617045 |
| PITPNM1 | 11 | 67491768..67505372 |  | ECT2 | 3 | 172750646..172829265 |
| ZNF432 | 19 | 52033424..52048820 |  | EIF4G1 | 3 | 184314495..184335358 |
| MATR3 | 5 | 139273752..139331677 |  | ACSL3 | 2 | 222861014..222943401 |
| OSBPL2 | 20 | 62238485..62296213 |  | ACSL4 | X | 109641335..109733392 |
| FIG4 | 6q21 | 110012424..110146634 |  | GAK | 4 | 849277..932390 |
| OPTN | 10p13 | 13142082..13180276 |  | GAPDH | 12 | 6534419..6538371 |
| UBE4B | 1 | 10032983..10181239 |  | GART | 21 | 33503931..33542917 |
| PAICS | 4 | 56410516..56461368 |  | GBA | 1 | 155204239..155214653 |
| RCAN3 | 1 | 24502351..24537020 |  | GCH1 | 14 | 54842005..54902885 |
| FAF1 | 1 | 50441263..50960264 |  | HSPA1L | 6 | 31809619..31821040 |
| PRAF2 | X | 49071156..49074045 |  | HSPA5 | 9 | 125234848..125241387 |
| RAB3GAP1 | 2 | 135052265..135176394 |  | HSPA8 | 11 | 123057492..123062335 |
| LSD1 | 1 | 23019443..23083691 |  | INSRR | 1 | 156840873..156858920 |
| WDTC1 | 1 | 27234516..27308633 |  | KCNA3 | 1 | 110653560..110675033 |
| SETX | 9 | 132261440..132356726 |  | RAB8A | 19 | 16111680..16133635 |
| VPS13A | 9 | 77177353..77417483 |  | NDUFV2 | 18 | 9102630..9134345 |
| BICD2 | 9 | 92711363..92764801 |  | NEDD4 | 15 | 55826917..55993746 |
| ARHGEF12 | 11 | 120336555..120489936 |  | PARK2 | 6 | 161768590..163148834 |
| PUM2 | 2 | 20248692..20350975 |  | PDE1C | 7 | 31752052..32299404 |
| TARDBP | 1p36.22 | 11072679..11085549 |  | PDE4B | 1 | 65792510..66374579 |
| PPP1R15A | 19 | 48872392..48876062 |  | PDE8A | 15 | 84980513..85139145 |
| OSBP2 | 22 | 30693782..30907824 |  | PPM1A | 14 | 60245752..60299087 |
| RAB3GAP2 | 1 | 220148268..220272501 |  | PPP1R3C | 10 | 91628440..91633101 |
| PAMR1 | 11 | 35431827..35530300 |  | PTPN9 | 15 | 75467121..75579291 |
| CHMP2B | 3 | 87227263..87255548 |  | RPS21 | 20 | 62387065..62388520 |
| OSBPL3 | 7 | 24796537..24980212 |  | SNCA | 4 | 90645250..90759447 |
| UBXN7 | 3 | 196353490..196432474 |  | TBCA | 5 | 77691170..77776361 |
| PABPC1 | 8 | 100702916..100722482 |  | UBE2D3 | 4 | 102794383..102868893 |
| UBQLN2 | Xp11.21 | 56590026..56593443 |  | UBE2E2 | 3 | 23203007..23590805 |
| SH3KBP1 | X | 19533965..19887626 |  | UBE2H | 7 | 129830732..129952960 |
| GPN3 | 12 | 110452486..110468829 |  | UCHL1 | 4 | 41256881..41268429 |
| VPS29 | 12 | 110491523..110502140 |  | UPP1 | 7 | 48088603..48108733 |
| TMEM106B | 7 | 12211203..12237264 |  | PLA2G6 | 22 | 38111495..38192051 |
| VPS13C | 15 | 61852389..62060465 |  | KLF11 | 2 | 10042993..10054836 |
| STX17 | 9 | 99906633..99974536 |  | RAB7L1 | 1 | 205737114..205744610 |
| FAM82A2 | 15 | 40735887..40755260 |  | PUM1 | 1 | 30931506..31065717 |
| LSG1 | 3 | 194640788..194672477 |  | NFAT5 | 16 | 69565966..69704666 |
| KIF16B | 20 | 16272097..16573434 |  | TREH | 11 | 118658232..118679672 |
| SPPL2B | 19 | 2328630..2355102 |  | PARK7 | 1 | 7961654..7985282 |
| MICAL3 | 22 | 17787649..18024559 |  | INPP5F | 10 | 119726047..119829151 |
| PITPNM2 | 12 | 122983480..123110488 |  | SREBF | 3 | 47413694..47476113 |
| ALS2 | 2 | 201700263..201781172 |  | SARM1 | 17 | 28371662..28401045 |
| HIVEP3 | 1 | 41506365..42035925 |  | NEDD4L | 18 | 58044355..58401540 |
| RNF123 | 3 | 49689517..49721529 |  | ATP13A2 | 1 | 16985958..17011972 |
| MRPL9 | 1 | 151759643..151763564 |  | SIRT1 | 10 | 67884669..67918390 |
| PINK1 | 1 | 20959948..20978004 |  | FBXO7 | 22 | 32474720..32498831 |
| IRX6 | 16 | 55324494..55330760 |  | RNF11 | 1 | 51236273..51273447 |
| USP38 | 4 | 143184917..143221988 |  | STK39 | 2 | 167954020..168247595 |
| GPNMB | 7 | 23246686..23275110 |  | PDE11A | 2 | 177623249..178108339 |
| OSBPL5 | 11 | 3087116..3165352 |  | SLC35C2 | 20 | 46347350..46364458 |
| LRRK2 | 12 | 40618813..40763087 |  | SIRT7 | 17 | 81911939..81918182 |
| PM20D1 | 1 | 205828022..205850148 |  | FBXW7 | 4 | 152321258..152535241 |
| YTHDF3 | 8 | 63168553..63212788 |  | VPS35 | 16 | 46659677..46689232 |
| MMP9 | 20 | 46008908..46016561 |  | MCCC1 | 3 | 183015218..183099585 |

**Supplemental Figure. Extended pedigree of *ATXN10* family**


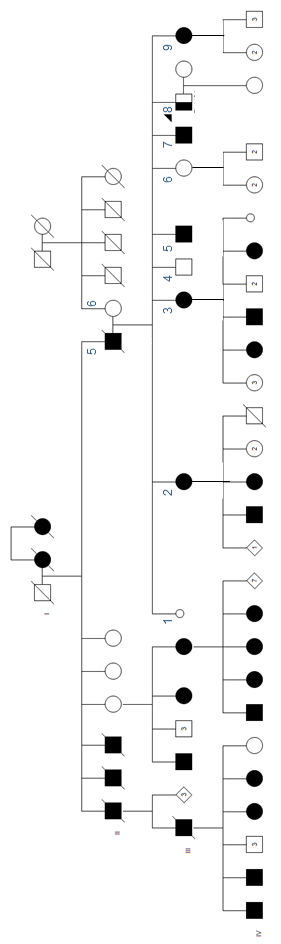

Supplement: Supplementary file 1 — Supplemental Material [file 41531_2017_29_MOESM1_ESM.docx]
